# Combination Therapy with Continuous Three-in-One Femoral Nerve Block and Periarticular Multimodal Drug Infiltration after Total Hip Arthroplasty

**DOI:** 10.1155/2016/1425201

**Published:** 2016-12-14

**Authors:** Tomonori Tetsunaga, Tomoko Tetsunaga, Kazuo Fujiwara, Hirosuke Endo, Toshifumi Ozaki

**Affiliations:** ^1^Department of Orthopaedics, Okayama University, 2-5-1 Shikata-cho, Kitaku, Okayama 700-8558, Japan; ^2^Department of Orthopaedics, Okayama Medical Center, 1711-1 Tamasu, Kitaku, Okayama 701-1192, Japan; ^3^Department of Intelligent Orthopedic Systems, Okayama University, 2-5-1 Shikata-cho, Kitaku, Okayama 700-8558, Japan

## Abstract

*Background*. Various postoperative pain relief modalities, including continuous femoral nerve block (CFNB), local infiltration analgesia (LIA), and combination therapy, have been reported for total knee arthroplasty. However, no studies have compared CFNB with LIA for total hip arthroplasty (THA). The aim of this study was to compare the efficacy of CFNB versus LIA after THA.* Methods*. We retrospectively reviewed the postoperative outcomes of 93 THA patients (20 men, 73 women; mean age 69.2 years). Patients were divided into three groups according to postoperative analgesic technique: CFNB, LIA, or combined CFNB+LIA. We measured the following postoperative outcome parameters: visual analog scale (VAS) for pain at rest, supplemental analgesia, side effects, mobilization, length of hospital stay, and Harris Hip Score (HHS).* Results*. The CFNB+LIA group had significantly lower VAS pain scores than the CFNB and LIA groups on postoperative day 1. There were no significant differences among the three groups in use of supplemental analgesia, side effects, mobilization, length of hospital stay, or HHS at 3 months after THA.* Conclusions*. Although there were no clinically significant differences in outcomes among the three groups, combination therapy with CFNB and LIA provided better pain relief after THA than CFNB or LIA alone, with few side effects.

## 1. Introduction

Acute postoperative pain is a distinct risk factor for prolonged pain [1]. Postoperative pain relief after joint surgery can be achieved with various modalities, such as patient-controlled analgesia (PCA) with morphine, epidural analgesia, and lumbar plexus and/or sciatic blocks [2–6]. The advantages of PCA include fewer technical problems than other modalities and uniform, sustained analgesia with autonomy [7, 8]. Although both PCA and continuous epidural analgesia provide sufficient pain relief, they are associated with multiple side effects, including arterial hypotension, respiratory depression, nausea/vomiting, and urinary retention [6]. Because the lumbar plexus is located in a deep tissue layer, lumbar plexus blockade is difficult and is associated with complications, including sensory nerve injury and retroperitoneal hemorrhage. Therefore, only experienced anesthesiologists should perform this procedure.

Local infiltration analgesia (LIA) offers better pain control, reduced narcotic consumption, and earlier mobilization, without increased risks [9]. Moreover, LIA reduces hospital stays compared with epidural anesthesia after total hip arthroplasty (THA) [10]. Continuous femoral nerve block (CFNB) is a popular analgesic modality after total knee arthroplasty (TKA), with fewer side effects than morphine or fentanyl [2, 6]. One study reported that CFNB was more effective than continuous epidural anesthesia or PCA after THA [11]. However, few studies have reported good outcomes with CFNB after THA [3, 6, 12]. The problem with using CFNB alone is that achieving adequate analgesia in the sciatic nerve region is difficult. Periarticular anesthetic infiltration offers a practical and potentially safer alternative to sciatic nerve block for patients undergoing TKA [13]. CFNB and LIA can complement one another. Koh et al. [14] compared combination therapy with CFNB and LIA with the use of CFNB alone and reported reduced pain with combination therapy in the initial 48 hours after TKA, along with reduced need for opioids in the initial 24 hours. However, the effects of combining CFNB and LIA on postoperative outcomes in patients undergoing THA have not been fully evaluated. The aim of the present study was to compare three-in-one CFNB, LIA, and the combination of CFNB and LIA on postoperative outcomes in patients after THA.

## 2. Methods

All patients included in this study gave their written, informed consent. Ethical approval was obtained from the Institutional Review Board of our institution. We retrospectively reviewed and analyzed the clinical outcomes of patients who received one of three postoperative analgesia protocols after THA. Inclusion criteria included patients who underwent THA at our hospital from January 2014 to March 2015 and who were able to provide informed consent and cooperate with the study. We excluded patients with local infection, cemented THA, revision THA, bleeding tendency due to anticoagulant therapy, renal insufficiency, or allergy to local anesthetics or other medications. This study included 93 patients (20 men and 73 women). The mean age at surgery was 69 years (range 37–92). The preoperative diagnoses were osteoarthritis in 81 hips, avascular necrosis of the femoral head in eight hips, rheumatoid arthritis in two hips, and femoral neck fracture in two hips. Three experienced surgeons performed all operations in this study. All THA surgeries were performed via the direct lateral (Hardinge) approach and used cementless implants (PINNACLE, TRILOCK; DePuy Synthes, Tokyo, Japan).

Anesthesiologists instituted one of the following analgesic modalities: CFNB (CFNB group, *n* = 30), LIA (LIA group, *n* = 32), or combined CFNB and LIA (CFNB+LIA group, *n* = 31) (Table 1). In the CFNB group, a continuous 3-in-1 block, which blocks the femoral nerve, obturator nerve, and lateral femoral cutaneous nerve, was administered after the induction of general anesthesia, according to the method described by Winnie et al. [15]. The femoral artery was located below the inguinal ligament; an 18-G needle (1.3 × 50-mm, Contiplex®; B-Braun, Tokyo, Japan) connected to a nerve stimulator (Stimuplex® HNS12; B-Braun, Tokyo, Japan) was inserted just lateral to the artery under ultrasound guidance (MICROMAXX®; SonoSite, Tokyo, Japan). The femoral nerve was accurately identified by eliciting contractions of the quadriceps with minimal stimulator settings (frequency, 2 Hz; current, 0.5 mA) [16]. Using the Seldinger technique, a 20-G catheter (0.85 × 1000 mm, Contiplex; B-Braun, Tokyo, Japan) was advanced 10 to 15 cm into the psoas compartment. After a negative aspiration test for blood, 0.2% ropivacaine (2 mg/ml, Anapeine®; AstraZeneca, Tokyo, Japan) was injected at a rate of 4 ml/h beginning immediately postoperatively. In the LIA group, 225 mg of 0.75% ropivacaine (7.5 mg/ml, Anapeine; AstraZeneca), 10 mg of morphine hydrochloride (10 mg/ml, Shionogi, Osaka, Japan), and 0.5 mg of epinephrine (1 : 1000) were mixed with 18.5 ml of sterile normal saline solution to make a combined volume of 50 ml, which was injected into the periarticular soft tissue. In the CFNB+LIA group, both a continuous 3-in-1 block and LIA were administered as described above. Breakthrough pain relief was achieved with a diclofenac sodium suppository (25 mg). Use of a diclofenac sodium suppository was allowed for all patients at any time after surgery; postoperative diclofenac sodium suppository use was assessed. Metoclopramide hydrochloride was used to treat postoperative nausea and vomiting (PONV).

On the first postoperative day, all patients started anticoagulation therapy with fondaparinux (2.5 mg/day), which was continued for 14 days. On the first postoperative day, all patients began a regimen of movement and strengthening exercises; they were encouraged to achieve full weight-bearing exercises on postoperative day 2.

### 2.1. Clinical Assessment

The primary outcome was patient pain intensity at rest on postoperative day 1. Early (postoperative days 1 to 7) walking capacity was assessed with straight-leg raising and T-cane ambulation. Middle-term walking capacity was measured 2 weeks after surgery with the 10-meter walking test and timed up and go test. Late-term physical activity was assessed 3 months after surgery with the Harris Hip Score (HHS) [17]. Food consumption, use of diclofenac sodium suppositories, side effects, and length of hospital stay were recorded for each group.

### 2.2. Pain Assessment

The patient's pain intensity at rest was self-assessed using a visual analog scale (VAS) 24 h postoperatively. The VAS for pain self-assessment is a widely used, valid, and reliable tool to measure pain intensity [18]. Patients rated pain intensity on a 100 mm VAS from no pain (0 mm) to unbearable pain (100 mm).

### 2.3. Ten-Meter Walking Test

The 10-meter walking test has been used in gait studies of patients with neurologic movement disorders in general [19] and to evaluate the spatial, temporal, and kinematic aspects of gait. This test measures the time it takes patients to walk 10 meters and assesses short-duration walking speed. Three trials were performed and the mean value was reported.

### 2.4. Timed Up and Go Test

Global muscle performance was assessed with the timed up and go test. First described by Podsiadlo and Richardson [20], this is a reliable and valid test that predicts the patient's ability to go outside alone safely. Subjects were instructed to sit in a chair at a height that maintained the hips and knees at 90° flexion, with their back very lightly touching the backrest. Stable posture was confirmed by the examiner. We observed and timed how long it took subjects to rise from the chair, walk 3 meters at a comfortable speed, turn, walk back, and sit down again. The complete sequence was measured three times, and the mean value was reported.

### 2.5. Statistical Analysis

Differences in data among groups were tested with one-way or two-way repeated-measures analysis of variance (ANOVA) with Tukey's post hoc test. The Pearson Chi-squared test was used to compare differences in sex ratio and diagnosis. A pilot study was performed with ten patients in each group. In the pilot study, the mean VAS was 30.5 mm in the CFNB group, 20.5 mm in the LIA group, and 13 mm in the CFNB+LIA group, with a standard deviation of 10. Based on the effect size in this pilot study, a power calculation for a trial (*p* < 0.05; power: 0.8) suggested that 29 patients would be needed in each group. Values are shown as mean ± standard deviation; values of *p* < 0.05 were considered significant. Statistical analysis was conducted with SPSS version 22 (IBM Corporation, Armonk, NY, USA) for Windows (Microsoft Corporation, Redmond, WA, USA).

## 3. Results

### 3.1. Analgesic Activity

**Figure 1:**
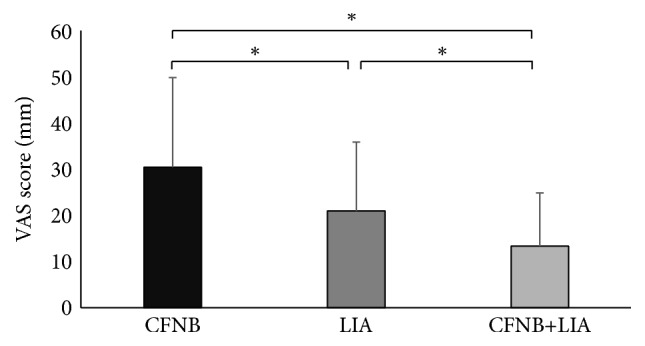
Visual analog scale (VAS) for pain at rest 24 hours postoperatively. Significant differences are seen among groups 24 hours after surgery. Data are presented as mean ± SD. CFNB, continuous femoral nerve block; LIA, local infiltration analgesia. *∗* indicates *p* < 0.05.

No significant differences in patient background were identified among groups. These data are summarized in Table 1. The VAS score at rest was 30.4 ± 19.4 in the CFNB group, 20.9 ± 14.9 in the LIA group, and 13.3 ± 11.5 in the CFNB+LIA group (Figure 1). Statistically significant differences were observed among the three groups in postoperative pain intensity at rest on the first postoperative day. The VAS score at rest in the CFNB+LIA group was significantly lower than those of the CFNB and LIA groups (*p* < 0.0001 and *p* = 0.04, resp.). The use of diclofenac sodium suppositories within 24 hours after surgery was similar among the three groups (*p* = 0.18, Table 2). Although three patients (8.6%) in the CFNB group, two (6.3%) in the LIA group, and three (9.7%) in the CFNB+LIA group had nausea/vomiting, patients consumed 71.8% of food offered on the first postoperative day. There were no significant differences in food consumption among groups on the first postoperative day (*p* = 0.56). The incidence of nausea/vomiting was not affected by the treatment modality (*p* = 0.88). None of the patients experienced systemic toxicity from ropivacaine. There were no cases of delayed wound healing, wound infection, or prosthesis infection. None of the patients required repeat surgery during the study period.

### 3.2. Mobilization

To evaluate early walking capacity, we assessed quadriceps strength and T-cane ambulation. Patients achieved straight-leg raise 5.6 ± 2.9 days after surgery and assisted ambulation with a walking cane 6.8 ± 2.6 days postoperatively. No significant differences were found among groups regarding either the straight-leg raise or T-cane ambulation (*p* = 0.69 and 0.51, resp.). Walking capacity was measured 2 weeks after surgery with the 10-meter walking test and timed up and go test. No significant differences were seen among groups in either test on postoperative day 14. The mean hospital stay after surgery, including postoperative physical therapy, was 19.2 ± 2.5 days. No significant differences in length of hospital stay were observed among the three groups (*p* = 0.48). The HHS was similar in the three groups 3 months after surgery (*p* = 0.90). HHS improved significantly in all three groups postoperatively (all *p* < 0.0001).

## 4. Discussion

This study compared the acute postoperative pain levels in patients receiving CFNB, LIA, or combined CFNB and LIA after THA. Our results indicate that combined CFNB and LIA provided significantly better pain relief on the first postoperative day than either modality alone. PONV and other perioperative complications were comparable among the three study groups. No significant differences were seen in patient mobilization among the three postoperative analgesia groups at 2 weeks or 3 months after surgery.

CFNB has been widely used in TKA patients [6, 21]. Compared with PCA and continual epidural anesthesia, CFNB provides good pain relief and is associated with fewer side effects such as PONV, hematomas, arterial hypotension, and drowsiness [3, 12]. However, few reports have analyzed CFNB in THA patients [3, 12]. Inadequate analgesia in the sciatic nerve region is the main reason CFNB is not widely used in THA. Although CFNB can be combined with a sciatic nerve block, periarticular anesthetic infiltration offers a practical and potentially safer alternative as an adjunct to CFNB in TKA patients [22].

In TKA, peri- and intra-articular infiltration of analgesics can improve early analgesia and mobilization more effectively than CFNB [23]. However, CFNB is associated with lower opioid consumption and better recovery at 6 weeks than periarticular infiltration [24]. Jiménez-Almonte et al. [25] reported no differences between LIA versus peripheral nerve blocks in analgesia or opioid consumption 24 hours after THA. Different protocols for femoral nerve blocks and LIA in these trials hinder interpretation and conclusions. There is strong evidence that pain at rest is lower in patients receiving LIA than in controls 24 hours after THA [26–28]. However, Marques et al. [28] reported that the effects of LIA alone without additional postclosure analgesia were limited to the first 24 hours after THA. Lunn et al. [29] reported the use of intraoperative high-volume LIA with 0.2% ropivacaine combined with a multimodal oral analgesic regimen consisting of acetaminophen, celecoxib, and gabapentin after THA, and use of LIA alone is not recommended. Given this information, we consider that additional postclosure analgesia including CFNB provides great value. In this study, we administered combined therapy with CFNB and LIA for the first time in patients after THA. After TKA, combined therapy with femoral nerve block and LIA has shown no evidence of added benefits for recovery of function [13]. Although no significant differences were seen in clinical function 3 months after surgery in the present study, acute pain was significantly lower with combination therapy. Reducing acute postoperative pain, which is a risk factor for chronic pain after THA, could lower the incidence of prolonged pain [1].

Because we found only slight differences among interventions, clinicians should focus on other factors such as cost and intervention-related complications when choosing analgesic treatments after THA [25]. A potential disadvantage of CFNB is an increased risk of falling due to quadriceps weakness. Ilfeld et al. [30] reported that 42% to 43% of patients experienced quadriceps femoris weakness at a dose of 0.2% ropivacaine at 8 ml/h; they recommended 0.2% ropivacaine at 3.0 ml/h in patients with quadriceps femoris weakness at 6.0 ml/h [31]. The concentration and flow rate of CFNB should be adjusted based on its effect [32]. In this study, no patients in the CFNB or CFNB+LIA groups complained of postoperative quadriceps weakness. We consider 0.2% ropivacaine at a rate of 4 ml/h to be appropriate to minimize the risk of falling after THA. To avoid dense sensory and motor blockade, a combination of an opioid and a local anesthetic is also recommended [7]. Opioid medication is a key strategy in the management of postsurgical pain but its associated PONV can delay mobilization and rehabilitation [11, 33]. Our infiltration analgesia injection contained 10 mg of morphine hydrochloride. The incidence of PONV was comparable with or without morphine in this study. Marques et al. [28] reported that patients receiving local anesthetic infiltration consumed 20% less morphine than patients receiving epidural analgesia and 12% less than patients receiving intrathecal analgesia. The lack of difference in PONV incidence among groups in our study might be explained by the low dose of morphine used. Neuropathy after peripheral nerve blockade is another complication of the analgesic procedure, estimated to occur in almost 3% of cases, although permanent injury is rare [34]. In the current study, there were no neurological complications after CFNB. The use of ultrasound with electrostimulation in this study enabled us to identify the femoral nerve and place the local block more precisely.

This study has some limitations. First, this is a retrospective study rather than a randomized-control trial. A prospective, randomized, controlled study should be conducted to eliminate selection bias and obtain more consolidated results. However, the present study had little selection bias because all surgeries were performed by experienced hip surgeons and there were no significant differences in patient backgrounds among groups. Second, we performed all THA under general anesthesia. Several clinical studies support the use of spinal anesthesia in orthopedic surgery [35–37]. Spinal anesthesia is associated with a lower complication rate [35], including reduced blood loss [36] and lower incidence of deep vein thrombosis [37]. We might have obtained different outcomes if we had used spinal anesthesia in the current study. Previous studies have reported shorter hospital stays among patients undergoing TKA with spinal anesthesia than among those receiving general anesthesia [38], making spinal anesthesia more cost-effective [39]. Third, the Japanese universal health insurance system complicates the analysis of length of hospital stay. Patients receiving LIA can reduce the length of hospital stay by adding analgesia through a catheter or injection [28]. In our study, no significant differences were seen in length of hospital stay among groups, because the Japanese universal health insurance system keeps medical expenses relatively low for all patients. Patients generally stay in the hospital until completion of their postoperative physical therapy. Finally, the results of this study apply only to patients who undergo THA via the direct lateral approach or modified Watson Jones approach; it is not possible to perform CFNB with a direct anterior approach because of the adjacent skin incision. In patients undergoing THA via a posterolateral approach, dividing short rotators and quadratus femoris, a different outcome might be obtained.

Three-in-one CFNB blocks the femoral nerve, obturator nerve, and lateral femoral cutaneous nerve without sciatic nerve blockade. LIA enables pain relief in accordance with the surgeon's demands, including the sciatic nerve region directly. In this study, we analyzed the additive effects of CFNB and LIA after THA. Although combination therapy with CFNB and LIA did not accelerate functional recovery in patients after THA, excellent pain relief with few side effects was achieved in patients following THA. Further studies with a larger sample size are needed to assess whether combination therapy with CFNB and LIA can prevent long-term pain.

## Figures and Tables

**Table 1:** Patient characteristics.

	Total	CFNB	LIA	CFNB+LIA	*p* value
	(*n* = 93)	(*n* = 30)	(*n* = 32)	(*n* = 31)	
Age (years)	69.2 ± 11.2	64 ± 10.4	66.6 ± 12.7	68 ± 11	0.25
Sex (male : female)	20 : 73	9 : 21	5 : 27	6 : 25	0.28
BMI (kg/m^2^)	24 ± 3.9	24 ± 4.3	23 ± 3.4	25 ± 3.8	0.19
Diagnosis					0.89
OA	81	28	27	26	
ANFH	8	2	3	3	
RA	2	0	1	1	
Fracture	2	0	1	1	
VAS (mm)	53.6 ± 22.2	50.5 ± 20.7	54.4 ± 21.3	56.1 ± 24.8	0.51
10 m test (s)	11.6 ± 4.2	11.5 ± 2.6	11.9 ± 3.9	11.5 ± 5.5	0.94
TUG (s)	12 ± 3.9	11.8 ± 2.7	12.8 ± 5.2	11.5 ± 3.6	0.48
HHS (points)	45.8 ± 16.3	45.5 ± 16.1	45.4 ± 14.1	42.6 ± 19.1	0.35
Operation time (min)	77 ± 25.3	81.1 ± 30.6	80.7 ± 19.7	79.5 ± 25	0.25
Blood loss (ml)	227 ± 183	252 ± 168	212 ± 219	217 ± 157	0.74

CFNB = continuous femoral nerve block; LIA = local anesthetic infiltration; BMI = body mass index; OA = osteoarthritis; ANFH = avascular necrosis of the femoral head; RA = rheumatoid arthritis; VAS = visual analogue scale; TUG = timed up and go test; HHS = Harris Hip Score.

**Table 2:** Overall comparison of outcomes.

	Total	CFNB	LIA	CFNB+LIA	*p* value
	(*n* = 93)	(*n* = 30)	(*n* = 32)	(*n* = 31)	
NSAIDs (*n* used/patient)	1.39 (0–4)	1.69 (0–4)	1.57 (0–4)	0.94 (0–3)	0.18
PONV	8 (8.6%)	3 (10%)	2 (6.3%)	3 (9.7%)	0.88
Meal (% consumed)	71.8 ± 23.4	74.2 ± 22.4	70.2 ± 25.2	71 ± 22.6	0.56
SLR achieved (day)	5.6 ± 2.9	6.1 ± 3.2	5.5 ± 2.7	5.2 ± 3.1	0.69
T cane walking (day)	6.8 ± 2.6	7.3 ± 3.0	6.6 ± 2.5	6.5 ± 2.4	0.51
10 m walking test (s)	14.2 ± 4.4	14.7 ± 4.6	14.4 ± 4.0	13.4 ± 4.7	0.67
TUG (s)	16.1 ± 5.2	16 ± 4.7	16.9 ± 5.6	15.2 ± 5.1	0.62
Hospital stay (days)	19.2 ± 2.5	19.7 ± 2.6	19 ± 2.4	19 ± 2.6	0.48
HHS (points)	83.2 ± 5.5	80.4 ± 14.9	80.4 ± 14.2	81.9 ± 14.6	0.90

CFNB = continuous femoral nerve block; LIA = local anaesthetic infiltration; VAS = visual analogue scale; NSAIDs = nonsteroid anti-inflammatory drugs; PONV = postoperative nausea and vomiting; SLR = straight leg raising; TUG = timed up and go; HHS = Harris Hip Score.
